# Topical Spray of dsRNA Induces Mortality and Inhibits Chilli Leaf Curl Virus Transmission by *Bemisia tabaci* Asia II 1

**DOI:** 10.3390/cells11050833

**Published:** 2022-02-28

**Authors:** Prosenjit Chakraborty, Amalendu Ghosh

**Affiliations:** Insect Vector Laboratory, Advanced Centre for Plant Virology, Indian Agricultural Research Institute, New Delhi 110012, India; nbu.prosen@yahoo.in

**Keywords:** silverleaf whitefly, begomovirus, RNAi, topical application, *heat shock protein 70*, *fasciclin 2*, virus transmission, virus-vector relationship

## Abstract

*Chilli leaf curl virus* (ChiLCV; genus: *Begomovirus*), transmitted *by Bemisia tabaci* (Gennadius) (Hemiptera: Aleyrodidae) in a persistent-circulative manner, is a major constraint in chilli production. The present study demonstrates for the first time that a topical spray of naked double-stranded RNA (dsRNA) on chilli plants causes mortality and inability to acquire and transmit ChiLCV in *B. tabaci*. dsRNA targeting *heat shock protein 70* (*hsp70*) and *fasciclin 2* (*fas2*) of *B. tabaci* Asia II 1 was first assessed under controlled conditions through oral delivery. *Hsp70* and *fas2* dsRNA resulted in up to 82.22% and 72% mortality of *B. tabaci* and around 12.4- and 8.5-fold decreases in mRNA levels, respectively, 24 h post-ingestion. ChiLCV copies in *hsp70* dsRNA-fed *B. tabaci* steadily decreased with an increase in dsRNA concentration and were undetectable at a higher concentration of dsRNA. However, ChiLCV copies significantly increased in *fas2* dsRNA-fed *B. tabaci*. Transmission of ChiLCV by *B. tabaci* was completely inhibited post-24 h feeding on *hsp70* dsRNA at 3 μg/mL. Naked *hsp70* dsRNA was topically sprayed on ChiLCV-infected chilli plants like an insecticide. 67.77% mortality of *B. tabaci*, 4.6-fold downregulation of *hsp70* mRNA, and 1.34 × 10^15^-fold decreased ChiLCV copies in *B. tabaci* were recorded when adults were exposed to the dsRNA-treated plants under semi-field conditions. Foliar application of naked dsRNA reduced the ChiLCV transmission by 75% without any visible symptoms in the inoculated plants. A total of 2 consecutive sprays of dsRNA provided significant protection to *B. tabaci* for up to 20 days under semi-field conditions.

## 1. Introduction

Silverleaf whitefly [*Bemisia tabaci* (Gennadius), Hemiptera: Aleyrodidae] is a phloem-feeding hemipteran insect that has been reported to infest over 600 plant species worldwide [1,2,3]. *B. tabaci* has been considered a complex of at least 46 morphologically indistinguishable cryptic species [4,5,6]. *B. tabaci* adults are about 1 mm long with small triangular-shaped bodies. The body is yellowish with distinct hyaline wings dusted with white powdery wax. Adults *B. tabaci* can be distinguished from other whiteflies by the position in which the wings are held over the body. The wings are held close to the body and tent-like in *B. tabaci*. It sucks phloem sap, leaving the affected plants extremely weak, and secretes a honeydew onto the surface of the leaves that promotes the growth of sooty mold fungus [7]. Besides direct damage caused by feeding, *B. tabaci* transmits more than a hundred begomoviruses, carlaviruses, criniviruses, cytorhabdoviruses, ipomoviruses, poleroviruses, and torradoviruses ([8,9,10,11,12,13,14,15,16]. Among them, begomoviruses represent 90% of the viruses transmitted by *B. tabaci*. Begomovirus species have become widespread in Central America, the Dominican Republic, Israel, Mexico, Trinidad, and across South East Asia, including Cambodia, Indonesia, India, and Thailand [17]. These viruses can cause an estimated yield loss of 50–90% in tomatoes and other crops, including beans, cassava, chilli, cotton, cucurbits, eggplant, papaya, and potatoes [18,19].

Chilli (*Capsicum annum* L., family Solanaceae) is one of the economically important crops produced in tropical and sub-tropical countries [20]. *Chilli leaf curl virus* (ChiLCV, genus *Begomovirus*, family *Geminiviridae*) is a major constraint of chilli production, causing annual losses of about USD 15 billion [21]. ChiLCV is a monopartite begomovirus that contains one circular, single-stranded DNA-A component of 2.7 kb in size. ChiLCV is transmitted by *B. tabaci* in a persistent-circulative manner. *Capsicum* spp. are the primary hosts for ChiLCV, but it also infects tomato and amaranth [22,23]. ChiLCV has been responsible for several epidemics in India and Sri Lanka [24,25]. The disease is typically manifested in the infected plants as upward curling, puckering, bunching of leaves, blistering of interveinal areas, thickening, and swelling of the veins, shortening of internodes and petioles, and stunting of the whole plant. The leaves become smaller and severely affected plants produce fewer and deformed fruits. Yield losses of 20–50% have been recorded in chilli [26], which may rise to 100% in the case of simultaneous infestation with thrips and mites [27]. Control options for ChiLCV and *B. tabaci* are very limited as insecticides continue to lose their efficacy due to the emergence of insecticide-resistant *B. tabaci* populations. Besides, insecticides adversely affect the environment and human health. Considering the continued lack of consumer acceptance, transgenic plant technology is not a feasible strategy. In recent years, RNA interference (RNAi) has shown promise in the management of insect pests and plant viruses. Double-stranded RNA (dsRNA), upon entering the host cell, is processed by the Dicer enzyme and, in association with RNA-induced silencing complex (RISC), cleaves the targeted mRNA [28]. Interrupting the interrelationship between *B. tabaci* and begomovirus using RNAi is a promising approach.. For successful persistent-circulative transmission, the begomovirus particles need to cross the midgut barrier of *B. tabaci* to circulate in hemolymph and reach the primary salivary glands [29,30]. Several proteins at the midguts of *B. tabaci*, such as heat shock proteins (Hsp), cyclophilins, peptidoglycan recognition protein, and a midgut protein, are known to interact with begomovirus coat protein (CP) for successful internalization [31,32,33,34]. RNA-Seq of *B. tabaci* in response to begomoviruses revealed significant upregulations of *hsp70*, *fasciclin 2* (*fas2*), and several other transcripts [31,35]. In gene regulatory network analyses, these genes were enriched with higher degrees of interactions. The same trend was recorded in the mRNA expression in reverse transcriptase quantitative real-time PCR (RT-qPCR) post-exposure to ChiLCV [35]. Although host cellular chaperones like Hsp70, along with other cochaperones play an important role in several cellular processes, host immunity, and stress responses [36], *hsp70* has been reported to be associated with begomovirus transmission by *B. tabaci* [31]. Neural cell adhesion molecule (NCAM) orthologues in *B. tabaci* like *fas2* function in synaptic development and growth [37,38]. Besides, NCAM molecules are known as receptors in virus replication [39]. The purpose of the present study is to validate the functions of *B. tabaci hsp70* and *fas2* in ChiLCV transmission using RNAi and explore the potentiality to use them as novel genetic tools for pest management.

Although several potential RNAi targets were identified for insects, including *B. tabaci* [40,41,42,43], most of those studies were conducted under controlled experimental conditions, and their efficacy under field conditions is limited. Rapid degradation of dsRNA in the extracellular environment and lack of reliable dsRNA delivery technique limits its applicability under field conditions. The objective of the present study was to induce resistance against *B. tabaci* and ChiLCV by spray-on application of dsRNA. In the present study, *B. tabaci hsp70* and *fas2* were silenced and resultant effects in mortality and virus transmission were reported. For the first time, the efficacy of spray-on application of naked dsRNA against a hemipteran insect was demonstrated under semi-field conditions. The outcomes of the study provide a novel, eco-friendly option for protection against *B. tabaci* as well as ChiLCV, which can reduce economic losses caused by the virus-vector complex. 

## 2. Materials and Methods

### 2.1. Whitefly Population

A homogeneous isofemale line of *B. tabaci* was raised from a single adult female and has been maintained on eggplants (var. Navkiran, Mahyco) at the Advanced Centre for Plant Virology, Indian Agricultural Research Institute (IARI), New Delhi, since 2015. The population was characterized by sequencing the mitochondrial cytochrome oxidase subunit I (mtCOI) gene (Table 1). The genotype or cryptic species of the *B. tabaci* population was confirmed based on Bayesian Inference phylogeny, considering a genetic divergence cutoff of 4% as described by Rehman et al. [6]. The population was maintained under controlled environmental conditions at 28 ± 2 °C, 60 ± 10% relative humidity, and a 16 h light-8 h dark photoperiod. 

### 2.2. Virus Culture

The inoculum of ChiLCV was collected from a pure culture maintained at the Advanced Centre for Plant Virology, IARI, New Delhi, India. ChiLCV was maintained in chilli (var. Preeti, Nunhems) by *B. tabaci* inoculation under insect-proof conditions. The identity of the virus was further confirmed by amplifying the DNA-A component in PCR with primer pairs Begomo F and Begomo R [44] (Table 1) and sequencing. 

### 2.3. Designing and Synthesis of dsRNA

For the selection of dsRNA fragments, complete gene sequences of *B. tabaci hsp70* (Accession Nos. HM367079, HM013712, EU934240, HM013709) and *fas2* (Accession No. XM_019049173) were downloaded from NCBI. The conserved sequences of the *hsp70* and *fas2* genes were analyzed in siRNA Wizard 3.1, Invivogen. The segments showing potential siRNA-forming regions were tested for cross-reactivity with other organisms like *Homo sapiens* (chromosomes, unplaced and unlocalized scaffolds), *Mus musculus* (chromosomes, unplaced and unlocalized scaffolds), *Aves* (taxid:8782), Lepidoptera (taxid:7088), Hymenoptera (taxid:7399), Formicidae (taxid:36668), and plants (taxid:3193) in NCBI using BLAST analysis. The region specific to *B. tabaci* with no cross-reactivity with other organisms was finally selected for synthesis of dsRNA. 

Primers targeting the dsRNA segment were designed in NCBI primer blast (https://www.ncbi.nlm.nih.gov/tools/primer-blast/ (Accessed on 4 June 2021) (Table 1). The primers AG137F-AG138R (for *hsp70*) and AG283F-AG284R (for *fas2*) were validated in a gradient PCR. The dsRNA stretches were amplified from isofemale *B. tabaci* Asia II 1 DNA in PCR with initial denaturation at 94 °C for 5 min, 30 cycles of 94 °C for 30 s, 56 °C for 30 s, and 72 °C for 30 s and a final extension step at 72 °C for 10 min. The PCR products were cloned into an L4440 plasmid vector between two T7 promoters. The recombinant plasmids were transformed into RNase III mutant *Escherichia coli* HT115 cells. Total RNA from the recombinant *E. coli* HT115 cells was extracted using Trizol reagent (Invitrogen, CA, USA) following the manufacturer’s protocol, and dsRNA was purified following Ahn et al. [45] with modifications. In brief, the recombinant *E. coli* HT115 cells were incubated for 12 h with continuous shaking at 37 °C followed by induction of T7 promoter with 1M isopropyl-β-D-1-thiogalactopyranoside (IPTG). The bacterial cells were harvested in a 1.5 mL microcentrifuge tube by centrifugation at 10,000× *g* for 5 min and resuspended in 1 mL Trizol. The cell suspension was mixed by vortexing and kept at room temperature for 5 min. The cell suspension was mixed with 200 μL chloroform, vortexed for <10 s, and incubated at room temperature for 10 min. The mixture was then centrifuged at 16,000× *g* for 10 min at 4 °C. The upper clear aqueous phase was transferred to a fresh tube and 0.8 volume of ice-chilled isopropanol was added. The solution was mixed properly and incubated at 4 °C for 10 min. The mixture was again centrifuged at 16,000× *g* for 10 min at 4 °C, and the supernatant was discarded. The pellet was washed with 70% ethanol, air-dried, and resuspended in 30 μL nuclease-free water. Total RNA isolated from recombinant *E. coli* HT115 cells was mixed with 1X RNA loading dye (Thermo Fisher Scientific, MA, USA), heated at 70 °C for 5 min, and visualized on 2% agarose gel stained with GoodView (BR Biochem, New Delhi, India). The total RNA was then treated with 1 unit of DNase I, RNase-free (Thermo Fisher Scientific) and 1 unit of RNase A, DNase-, and protease-free (Thermo Fisher Scientific) in the presence of 500 mM sodium chloride and incubated for 1 hr at 37 °C to eliminate the DNA and single-stranded RNA contaminants. The enzymes were inactivated by chloroform extraction, and the remaining dsRNA was resuspended in nuclease-free water. The purified dsRNA was quantified in a spectrophotometer (NanoDrop 2000, Thermo Fisher Scientific), and the integrity of the specific dsRNA was confirmed on 2% native agarose gel stained with GoodView and visualized in a gel documentation system (Maestrogen, Xiangshan District, Taiwan). 

**Table 1:** Primers used in the study.

Primer Name	Primer Sequence (5’-3’)	Amplicon Size	Annealing Temperature in PCR/Real-Time PCR	Amplified Region	Purpose	Reference
C1-J-2195	TTGATTTTTTGGTCATCCAGAAGT	860 bp	53 °C	*B. tabaci mtCOI*	Detection of *B. tabaci* cryptic species	[46]
L2-N-3014	TCCAATGCACTAATCTGCCATATTA					
Begomo F	ACGCGTGCCGTGCTGCTGCCCCCATTGTCC	2.7 kb	57 °C	*Begomovirus* DNA-A	Detection of begomovirus	[44]
Begomo R	ACGCGTATGGGCTGYCGAAGTTSAGAC					
AG137F	TCAAAGAACATTTTTGTGCTACT	128 bp	56 °C	*B. tabaci hsp70* dsRNA	dsRNA synthesis	This study
AG138R	GACCATTGTCTAGGTCTTCATTT					
AG283F	CTGGTGTTTTGACAATCGAC	150 bp	56 °C	*B. tabaci fas2* dsRNA	dsRNA synthesis and RT-qPCR	This study
AG284R	TGATTATGCCTTCTTCCGTC					
AG177F	ACATGGAAAAGATCTGGCAT	121 bp	56 °C	*B. tabaci β-actin*	RT-qPCR	This study
AG178R	TGAGTCATCTTTTCACGGTT					
AG204F	GTCAATGATTGCAGTAAGCC	105 bp	56 °C	*B. tabaci hsp70*	RT-qPCR	This study
AG205R	TTCCCTCATTTTCGTAAGCA					
AG149F	TGAACAGGCCCATGAACAG	290 bp	53 °C	ChiLCV coat protein	qPCR and ChiLCV detection	[47]
AG150R	ACGGACAAGGAAAAACATCAC					

### 2.4. Bioassay of dsRNA through Artificial Feeding under Controlled Conditions 

The purified *hsp70* and *fas2* dsRNA were individually and orally delivered to *B. tabaci* adults following the method of Upadhyay et al. [40] with modifications. In brief, an artificial diet was prepared by mixing 20% sucrose and 5% yeast extract in sterile distilled water and autoclaved. The artificial diet was supplemented with 1.0, 2.0, and 3.0 μg/mL of dsRNA and sandwiched between 2 layers of UV-sterilized stretched Parafilm M membranes on the open mouth of a 15 mL cylindrical pet bottle (3.5 cm diameter and 16 cm height). A hole was made on the wall of the bottle and covered with muslin cloth for ventilation. A batch of 30 adults of *B. tabaci* was released in each bottle to feed on the dsRNA mixed with the artificial diet for 24 h. Sterile distilled water instead of dsRNA was served as control. After 24 h of feeding, percent mortality was calculated and compared with untreated control. Three replicates for each concentration were maintained and repeated twice. The mean mortalities in each treatment were corrected by normalizing mortality in the control set. Mean differences among the categories were separated by Tukey’s test at a confidence interval of 95% using XLSTAT 2014.5.03. The surviving *B. tabaci* from several such replicates were used for determining the relative expression of *hsp70* and *fas2* mRNA and assessing the ChiLCV acquisition and transmission efficiency post-dsRNA feeding as described below.

### 2.5. Estimating Relative Expression of hsp70 and fas2 mRNA

Relative expression of *B. tabaci hsp70* mRNA post-dsRNA exposure was estimated by RT-qPCR assay following the 2^−ΔΔC^_T_ method [48]. The *β-actin* gene served as endogenous control. The primer pairs (AG204F-AG205R for *hsp70*; AG283F-AG284R for *fas2*; and AG177F-AG178R for *β-actin*) used in RT-qPCR are listed in Table 1. After 24 h of *has70* and *fas2* dsRNA feeding at 1.0, 2.0, and 3.0 μg/mL as described above, the surviving *B. tabaci* were collected from each of the treatments separately. About 30 surviving *B. tabaci* in 3 replicates were used for each of the treatments and doses for estimating the relative expression of the target genes. The *B. tabaci* adults were crushed in 1 mL Trizol reagent within microcentrifuge tubes using a hand automizer. Total RNA was isolated as described earlier. Total RNA was quantified in a spectrophotometer (NanoDrop 2000, Thermo Fisher Scientific), and complementary DNA was synthesized using the FIREScript RT cDNA synthesis kit (Solis BioDyne, Tartu, Estonia) with 1.0 μg template RNA for each set. The reaction mixture contained 1X RT reaction buffer, 1.0 μg template RNA, 5.0 μM oligo dT primer, 500 μM dNTP mix, 10 units of FIREScript RT, and 1 unit of RiboGrip RNase inhibitor. The reverse transcription was carried out in a thermocycler (T100, Bio-Rad, CA, USA) at 42 °C for 60 min, followed by enzyme inactivation at 85 °C for 5 min. The relative RT-qPCR assay was carried out in an Insta Q48M real-time PCR (Himedia, Mumbai, India) with 20 μL reaction mixture containing 10 μL of 1X Maxima SYBR green master mix, 10 μM ROX passive reference dye, 10 pmole each forward and reverse primer (AG204F-AG205R for *hsp70*; AG283F-AG284R for *fas2*; and AG177F-AG178R for *β-actin*), and 2 μL template cDNA. Thermal cycling was performed as initial denaturation at 94 °C for 5 min, 30 cycles of 94 °C for 30 sec, 56 °C for 30 s, and 72 °C for 30 s. Since SYBR Green I dye binds non-specifically to any double-stranded DNA, a dissociation or melting stage was carried out after every reaction to determine the specificity of the amplicons based on the melting curve. The RT-qPCR was performed with three biological and two technical replicates. The fold change in expression was normalized by excluding the changes in cycle threshold (C_T_) value of the endogenous control, *β-actin*. Log_2_ fold change value was calculated, and relative expression of mRNA was determined by normalizing the log_2_ 2^−ΔΔC^_T_ values of the dsRNA-treated samples with untreated control [48]. Statistical analysis and preparation of graphs were carried out in Microsoft Excel 2016.

### 2.6. Quantification of Virus Copies in B. tabaci and Transmission of ChiLCV

A portion of *B. tabaci* that survived post-feeding of dsRNA at 1.0, 2.0, and 3.0 μg/mL as described above was allowed to feed on ChiLCV-infected chilli plants (var. Preeti) for 24 h. About 50 surviving *B. tabaci* in 3 replicates were used for each of the treatments and doses. ChiLCV copies acquired after 24 h feeding by dsRNA-exposed *B. tabaci* were estimated by absolute quantification in qPCR. A standard curve of ChiLCV was prepared using a clone of partial ChiLCV CP gene in a pJET1.0 vector (Thermo Fisher Scientific). Then, 10-fold serial dilutions (5 × 10^2^ to 5 × 10^−5^ ng) of linearized plasmid were amplified in qPCR as described below. The standard curve was prepared by plotting a linear regression curve with log_10_ DNA dilutions on the *X*-axis and C_T_ values on the *Y*-axis. Three replicates of each dilution were used in preparing the standard curve. The statistical analysis and preparation of graphs were carried out in Microsoft Excel 2016.

After 24 h of acquisition, DNA was isolated using CTAB extraction buffer [49] from *B. tabaci* (30 per replicate) exposed to different doses (1.0, 2.0, and 3.0 μg/mL) of *hsp70* and *fas2* dsRNA separately. Briefly, *B. tabaci* adults were crushed in 1 mL CTAB extraction buffer (2% CTAB, 100 mM Tris-HCl pH 8.0, 20 mM EDTA pH 8.0, 1.4 M NaCl, and 2 μL β-mercaptoethanol). The homogenate was heated in a dry bath at 65 °C for 30 min, and an equal volume of chloroform-isoamyl alcohol (24:1) was added to it. The mixture was vortexed for <10 s, incubated at room temperature, and centrifuged at 16,000× *g* for 15 min. The upper aqueous phase was transferred to a fresh microcentrifuge tube, and an equal volume of isopropanol was added to precipitate the DNA. The solution was then centrifuged at 16,000× *g* for 15 min to pellet down the DNA and washed with 70% ethanol. The pellet was air-dried and dissolved in 30 μL sterile distilled water followed by qPCR with the ChiLCV-specific primers AG149F and AG150R [47] (Table 1). The qPCR was carried out in an Insta Q48M real-time PCR with a 20 μL reaction mixture as described above. Thermal cycling was performed as initial denaturation at 94 °C for 5 min, 30 cycles of 94 °C for 30 s, 53 °C for 30 s, and 72 °C for 30 s. Melting curve analysis was carried out after each reaction to check the specificity of the amplicons. Each treatment was comprised of three biological and two technical replicates.

The mean C_T_ values obtained in qPCR were fitted into the standard curve, and the resulting concentration was used for the calculation of virus copy number in Microsoft Excel 2016 using the following formula. Mean differences among the mean virus copies were separated by Tukey’s test at a confidence interval of 95% using XLSTAT 2014.5.03.

$$Virus\;copy\;number\;\left(N\right)=\frac{\left(x\times 6.022\times 10^{23}\right)}{\left(n\times 660\times 10^{9}\right)}\;$$

where *N* = number of viral copies, *x* = amount of amplicon in ng, and *n* = length of linearized plasmid DNA. 

For the ChiLCV transmission experiment, *B. tabaci* surviving different doses (1.0, 2.0, and 3.0 μg/mL) of *hsp70* and *fas2* dsRNA exposure were allowed to feed on ChiLCV-infected chilli plants for 24 h. After acquisition feeding, they were released onto healthy chilli plants (var. Preeti) at the 4–6 leaf stage for 24 h of inoculation feeding and eliminated thereafter. Two adult females per plant were released. All the plants were maintained under insect-proof conditions and monitored for symptom development. ChiLCV infection in inoculated plants was confirmed by PCR 35 days post-inoculation. The transmission efficiency was calculated as the percent of plants infected by ChiLCV post-24 h inoculation feeding by dsRNA-treated *B. tabaci*. *B. tabaci* without any exposure to dsRNA was used as a control. For each treatment, three biological replicates were used, and each replicate contained five plants. Tukey’s test was used to test significant differences in transmission efficiency. *p*-values less than 0.05 were considered statistically significant.

### 2.7. Topical Spray of hsp70 dsRNA under Contained Semi-Field Conditions 

A 10-fold higher dose of dsRNA (30 μg/mL) than the controlled condition assay was used to test its efficacy in a contained experiment under semi-field conditions where plants were covered by insect-proof nets and kept in the open air. The dsRNA was not artificially fed or applied directly on *B. tabaci*. Instead, naked *hsp70* dsRNA was topically sprayed on the ChiLCV-infected chilli plants (var. Preeti) like an insecticide in an insect-proof net house. Sterile distilled water was sprayed as a control. The plants were allowed to air-dry, and 100 virus-free *B. tabaci* adults were released on each dsRNA-treated ChiLCV-infected plant after 24 h of dsRNA spray. A total of 3 biological replicates, each containing 10 plants, were maintained. Percent mortality of *B. tabaci* 24 h after release on dsRNA-treated plants was calculated by normalizing the mean mortality in the control set. About 30 surviving *B. tabaci* in 3 replicates were used for determining the *hsp70* mRNA levels in RT-qPCR as described above. The other portion of surviving *B. tabaci* was used for transmission of ChiLCV and was released onto 4–6 leaf stage healthy chilli plants (5 adults/plant) for 24 h of inoculation feeding. All the plants were maintained in insect-proof conditions and tested in PCR for ChiLCV-infection 35 days post-inoculation. A total of 5 biological replicates containing 12 plants each were used, and transmission efficiency was calculated as the percent of plants infected by ChiLCV. Tukey’s test was performed to determine the significant differences in mean mortality and transmission efficiency among the categories at a confidence interval of 95%.

### 2.8. Stability of hsp70 dsRNA in Leaf Tissue

To assess the stability of the dsRNA on the topically sprayed plants, apical leaf tissue was collected at 1, 3, 6, 24, and 48 h post-dsRNA spray in 3 replicates. Plants sprayed with sterile distilled water served as control. Total RNA was isolated from 100 mg of plant tissues using Trizol reagent as described above. cDNA was synthesized using *hsp70* dsRNA-specific primer (AG137F and AG138R), and the presence of *hsp70* dsRNA was checked in PCR as described above. The experiment was repeated twice.

### 2.9. Persistent Efficacy of hsp70 dsRNA to Eradicate B. tabaci

The persistency in eradicating *B. tabaci* population by topical spray of *hsp70* dsRNA was evaluated in a contained experiment under semi-field conditions. Chilli plants were topically sprayed with *hsp70* dsRNA at 30 μg/mL. *B. tabaci* were released on the dsRNA-treated plants after 24 h of dsRNA spray. At every 24 h, old *B. tabaci* adults were manually eliminated, and fresh adults were released to check the persistency in the efficacy of dsRNA. A total of 30 adults per plant were released each and every time, and percent mortality was recorded at 24 h post each release. This was continued until the mortality percentage decreased significantly. Upon significant decrease in mortality after the first spray, the second application of dsRNA was undertaken. The mortality percentage was recorded at 24 h intervals after the second spray, as described above. Sterile distilled water was sprayed in place of dsRNA as untreated control. Three biological replicates, each containing five plants, were used to calculate the percent mortality. 

## 3. Results 

### 3.1. Characterization of B. tabaci and Begomovirus 

A homogeneous population of *B. tabaci* developed from a single adult female was characterized by sequencing the mtCOI gene. PCR amplification of *B. tabaci* mtCOI with C1-J-2195 and L2-N-3014 primers (Table 1) showed an expected amplicon of ~860 bp on an agarose gel. The nucleotide (nt) sequence showed 99.99% homology in BLASTn analysis with other *B. tabaci* Asia II 1 sequences in NCBI. The sequence can be retrieved using the GenBank Accession No. MT920041. Bayesian inference phylogeny considering genetic divergence cutoff of 4% revealed that the population belonged to the cryptic species *B. tabaci* Asia II 1 (data not presented). 

The identity of the virus was confirmed by sequencing the DNA-A component from infected chilli plants. PCR amplification of the DNA-A using Begomo F and Begomo R primer produced a 2.7 kb product visualized on 1% agarose gel. Bidirectional sequencing of the cloned products produced a 2763 nt sequence comprising complete DNA-A that showed 100% nucleotide identity to ChiLCV isolates upon BLASTn analysis. The sequence can be retrieved by GenBank Accession No. OM513903.

### 3.2. Synthesis of dsRNA Targeting B. tabaci hsp70 and fas2

**Figure 1:**
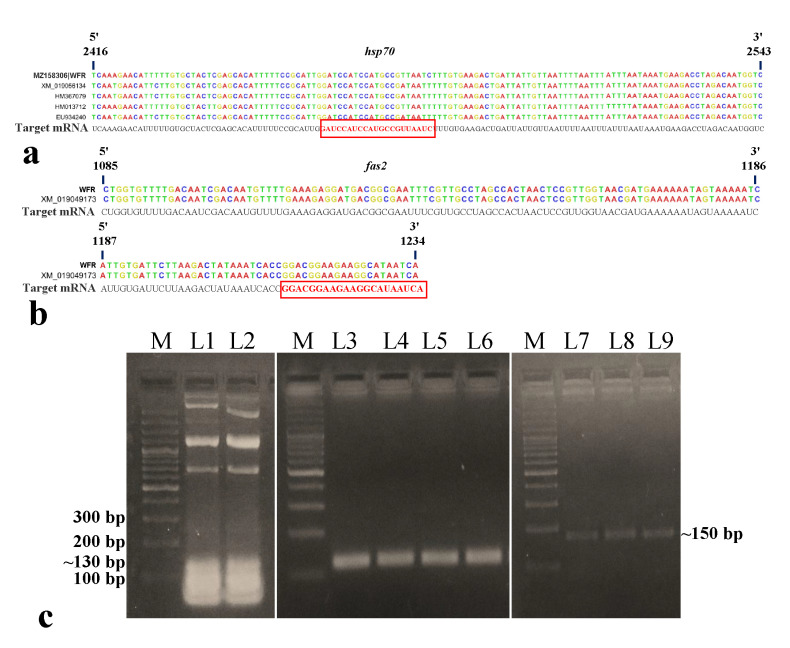
Designing and synthesis of dsRNA targeting *Bemisia tabaci hsp70* and *fas2* mRNA. (**a**) A conserved region of 128 (2416 to 2543) nt of *B. tabaci hsp70* was selected for designing dsRNA. The putative siRNA is marked within the red box. (**b**) A conserved region of 150 (1085 to 1234) nt of *B. tabaci fas2* was selected for designing dsRNA. The putative siRNA is marked within the red box. (**c**) Total RNA isolated from recombinant *E. coli* HT115 cells (L1–2); *hsp70* dsRNA purified from total RNA using DNase I and RNase A (L3–6); and *fas2* dsRNA purified from total RNA using DNase I and RNase A (L7–9) on 2% agarose gel stained with GoodView, M = 100 bp plus ladder.

A conserved 128 nt-long (2416 to 2543 nt) fragment of the *hsp70* gene (~2.5 kb) and a 150 nt-long (1085 to 1234 nt) fragment of the *fas2* gene (~1.4 kb) of *B. tabaci* were selected for dsRNA designing (Figure 1a,b). In siRNA Wizard 3.1, the 128 nt-long *B. tabaci hsp70* fragment produced a putative siRNA of 21 nt (5’-GAUCCAUCCAUGCCGUUAAUC-3’). The 150 nt-long *fas2* fragment also yielded one putative siRNA of 21 nt (5’-GGACGGAAGAAGGCAUAAUCA-3’). The dsRNA sequence was specific to *B. tabaci,* and no cross-reactivity was recorded with other organisms such as *Homo sapiens*, *Mus musculus*, *Aves* (taxid:8782), Lepidoptera (taxid:7088), Hymenoptera (taxid:7399), Formicidae (taxid:36668), and plants (taxid:3193) in BLAST analysis. PCR amplification of targeted *B. tabaci hsp70* and *fas2* fragments with AG137F-AG138R and AG283F-AG284R primer pairs gave single expected amplicons of ~130 bp and ~150 bp, respectively. The fragments were cloned between two T7 promoters of L4440 RNAi vector (Addgene 1654 provided by Andrew Fire, Carnegie Institution for Science, Washington, DC, USA). The recombinant plasmids were transformed into RNase III mutant *Escherichia coli* HT115 cells (provided by Caenorhabditis Genetics Center, Minneapolis, MN, USA). Total RNA isolated from recombinant *E. coli* HT115 cells and the dsRNA purified from total RNA using DNase I and RNase A were visualized in agarose gel electrophoresis (Figure 1c). The purified dsRNA of *hsp70* and *fas2* produced single specific bands of ~130 bp and ~150 bp on 2% native agarose gel (Figure 1c). Upon bidirectional sequencing of the recombinant plasmids, both the sequences of *hsp70* (Accession No. MZ158306) and *fas2* (Accession No. MZ766125) showed 100% homology with *B. tabaci hsp70* and *fas2*, respectively. 

### 3.3. Effect of dsRNA on Mortality and mRNA Expression of B. tabaci under Controlled Conditions

**Figure 2:**
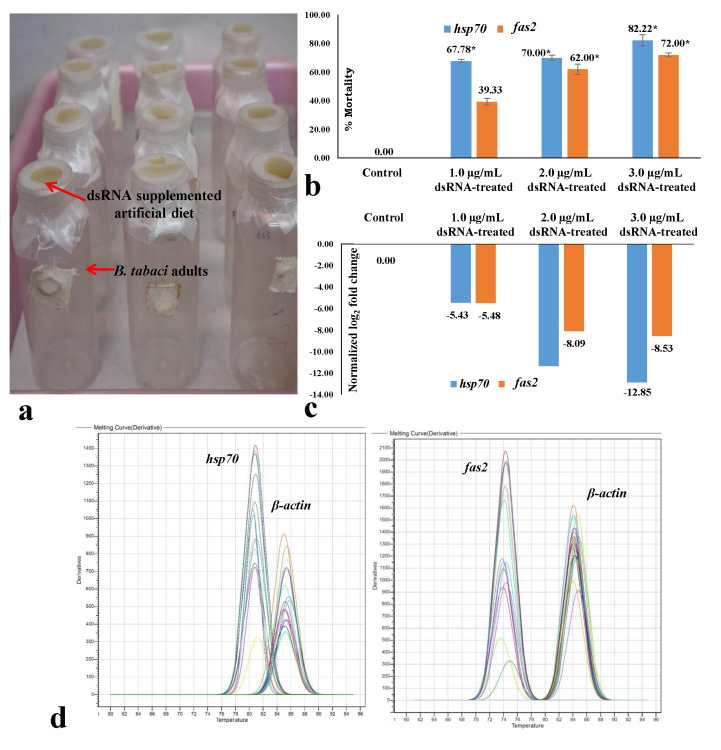
Effect of *hsp70* and *fas2* dsRNA delivered to *Bemisia tabaci* through artificial diet. (**a**) Artificial feeding setup for *B. tabaci* adults. The artificial diet was supplemented with different concentrations of dsRNA and sterile water (control). (**b**) Percent mortality of adult *B. tabaci* after 24 h of *hsp70* and *fas2* dsRNA feeding at 1.0, 2.0, and 3.0 μg/mL in comparison to control. Values above each column are means of three replications. The experiment was repeated twice. The mean mortality was calculated by normalizing the mean mortality in the control set. The error bars are standard error of the mean (SEM). Mean denoted by an asterisk (*) indicates a significant difference (*p*  <  0.05). (**c**) Normalized relative expression of *B. tabaci hsp70* and *fas2* mRNA after 24 h of dsRNA feeding in comparison to control. Relative quantification was done with respect to an endogenous control gene, *β-actin*. (**d**) Melting (dissociation) curves of *hsp70*, *fas2*, and *β-actin* in RT-qPCR. The specific peaks of *hsp70, fas2*, and *β-actin* amplicons without any secondary peaks indicated the specificity of the reactions.

The efficacy of *hsp70* and *fas2* dsRNA was first evaluated under controlled laboratory conditions. Significantly higher mortality of *B. tabaci* adults was recorded in *hsp70* dsRNA than *fas2* dsRNA when orally delivered under controlled laboratory conditions (Figure 2a). Mortality of 67.77% of *B. tabaci* was observed at 24 h upon feeding on 1.0 μg/mL *hsp70* dsRNA. The mortality of *B. tabaci* significantly increased with an increase in dsRNA concentration. The mean mortality was 70.0% when *hsp70* dsRNA was fed to *B. tabaci* adults at 2.0 μg/mL, and it increased significantly up to 82.22% at 3.0 μg/mL *hsp70* dsRNA (Figure 2b). Oral delivery of *fas2* dsRNA at 1.0 μg/mL exhibited 39.33% mean mortality after 24 h (Figure 2b). Significantly higher mortality (62 and 72%) of *B. tabaci* adults was recorded when 2.0 and 3.0 μg/mL *fas2* dsRNA was orally delivered to *B. tabaci* under controlled laboratory conditions. At all doses, the mortality of *B. tabaci* post-*fas2* dsRNA exposure was significantly lower than the *hsp70* dsRNA at *p*  <  0.05. However, no morpho-deformities were observed in killed *B. tabaci* adults either with *hsp70* or *fas2* dsRNA. 

RT-qPCR analysis showed that *hsp70* dsRNA significantly reduced the *hsp70* mRNA expression levels in treated *B. tabaci*. *Hsp70* mRNA levels in adult *B. tabaci* were decreased by 5.43-fold with respect to an endogenous control gene, *β-actin,* post-24 h feeding on *hsp70* dsRNA (Figure 2c). The mRNA expression levels were significantly reduced with an increase in *hsp70* dsRNA concentration. An exposure to *hsp70* dsRNA at 2.0 μg/mL for 24 h significantly reduced the *hsp70* mRNA expression by 11.32-fold and up to 12.85-fold when fed with 3.0 μg/mL *hsp70* dsRNA. The *fas2* mRNA expression levels were declined by 5.48-fold post-24 h feeding on *fas2* dsRNA at 1.0 μg/mL. The mRNA expression levels post-*hsp70* and -*fas2* dsRNA feeding were almost similar at 1.0 μg/mL. The mRNA expression levels were significantly reduced up to 8.53-fold with an increase in *fas2* dsRNA concentration. The reduction in mRNA expression level was significantly higher post-*hsp70* dsRNA feeding compared to *fas2* dsRNA at 2.0 and 3.0 μg/mL. The primer pairs for *hsp70*, *fas2*, and *β-actin* did not produce any secondary peaks in the RT-qPCR melting curve analysis that indicated the specificity of the reactions (Figure 2d,e).

### 3.4. Effect of dsRNA on Virus Acquisition and Transmission by B. tabaci under Controlled Conditions 

**Figure 3:**
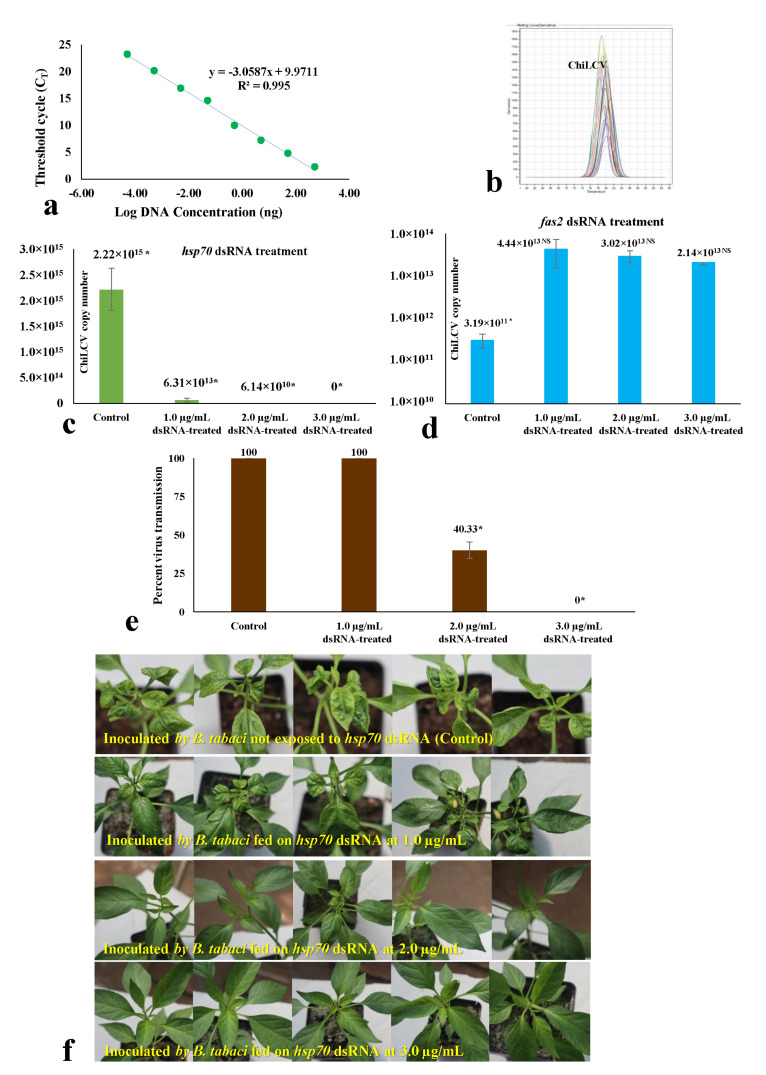
Effects of *hsp70* and *fas2* dsRNA in relation to ChiLCV acquisition by *B. tabaci* and respective involvement of *hsp70* dsRNA in transmission. (**a**) Standard curve of ChiLCV shows a linear relationship between log DNA concentrations in ng on the *X*-axis and × values on the *Y*-axis. Each concentration was replicated thrice. The equation of the straight line and the coefficient of correlation (R^2^) are mentioned on the graph. (**b**) Melt curves of ChiLCV amplicon in qPCR analysis. A single specific peak at 81 °C indicates the specificity of the reactions. (**c**) Mean ChiLCV copy numbers retained by *hsp70* dsRNA-treated *B. tabaci* after 24 h of acquisition feeding on ChiLCV-infected plants. (**d**) Mean ChiLCV copy numbers retained by *fas2* dsRNA-treated *B. tabaci* after 24 h of acquisition feeding on ChiLCV-infected plants. The error bars are standard error of the mean (SEM). Mean denoted by an asterisk (*) indicates a significant difference (*p*  <  0.05). (**e**) Percent transmission of ChiLCV from infected to healthy chilli plants by *hsp70* dsRNA-exposed *B. tabaci*. (**f**) Leaf curl symptoms on chilli (var. Preeti) plants 42 days after ChiLCV inoculation by *hsp70* dsRNA-fed *B. tabaci*. All the control plants produced typical leaf curl and vein clearing symptoms. All the plants inoculated by *B. tabaci* fed with 1.0 μg/mL *hsp70* dsRNA were ChiLCV-positive in PCR but produced less severe symptoms of leaf curl than control. The transmission efficiency of *B. tabaci* fed with 2.0 μg/mL *hsp70* dsRNA was 40%; inoculated plants produced mild leaf curl symptoms. All the plants inoculated by *B. tabaci* fed with 3.0 μg/mL *hsp70* dsRNA did not produce any symptoms and were ChiLCV-negative in PCR. For each treatment, three biological replicates were used and each replicate contained five plants.

After 24 h of dsRNA feeding, the adult *B. tabaci* were allowed to feed on ChiLCV-infected plants for 24 h. A standard curve of ChiLCV was prepared in qPCR using ten-fold dilutions of a clone of the partial ChiLCV CP gene. The standard curve of ChiLCV showed a coefficient of correlation (R^2^) of 0.995 and high amplification efficiency near to 100%, indicating optimal conditions for absolute quantification (Figure 3a). The virus copies acquired by the *B. tabaci* were quantified by fitting the mean C_T_ values obtained in qPCR into the standard curve. The specificity of the reactions was confirmed by a single peak at 81 °C in the melting curve analysis of qPCR (Figure 3b). The virus copy number acquired by *hsp70* dsRNA-treated *B. tabaci* was significantly less than the untreated control. When *B. tabaci* was fed with sterile distilled water instead of *hsp70* dsRNA, the mean ChiLCV copy number was 2.22 × 10^15^, whereas it decreased by 35.18-fold (6.31 × 10^13^ copies) post-exposure to *hsp70* dsRNA at 1.0 μg/mL (Figure 3c). The virus copy number in treated *B. tabaci* further decreased with a gradual increase in *hsp70* dsRNA concentration. At 2.0 μg/mL *hsp70* dsRNA, ChiLCV copy numbers were 6.14 × 10^10^ in *B. tabaci* i.e., 3.62 × 10^4^-fold lower than the untreated control. The virus copies were undetectable in *B. tabaci* with a further increase in *hsp70* dsRNA concentration to 3.0 μg/mL. The retention of ChiLCV might be completely ceased at a higher concentration of *hsp70* dsRNA or beyond the detection limit using qPCR. In contrast, the ChiLCV copy number acquired by *fas2* dsRNA-treated *B. tabaci* was significantly higher than the untreated control (Figure 3d). When *B. tabaci* was fed with sterile distilled water instead of *fas2* dsRNA, the mean ChiLCV copy number was 3.19 × 10^11^, whereas the virus copies increased significantly by 139.18-fold (4.44 × 10^13^ copies) at 1.0 μg/mL of *fas2* dsRNA feeding. However, the mean virus copies were estimated to be 3.02 × 10^13^ and 2.14 × 10^13^ after exposure to 2.0 and 3.0 μg/mL *fas2* dsRNA, respectively. Although significantly higher numbers of virus copies were recorded in all *fas2* dsRNA doses compared to the control, higher doses of *fas2* dsRNA recorded a decline in virus copies compared to the lower doses. As the virus copies in *B. tabaci* increased post-*fas2* dsRNA feeding, it was not assessed further for inhibiting transmission of ChiLCV and topical application. 

A similar pattern was recorded in the transmission of ChiLCV from infected to healthy chilli plants by *hsp70* dsRNA-fed *B. tabaci*. A 100% transmission of ChiLCV was recorded when *B. tabaci* was not exposed to *hsp70* dsRNA in the control set. All the inoculated plants produced strong leaf curl and vein-clearing symptoms and tested ChiLCV positive in PCR. Although there was a significant decrease in ChiLCV copies in *B. tabaci* exposed to 1.0 μg/mL *hsp70* dsRNA, all the test plants were found to be infected with ChiLCV in PCR testing at 35 days post-inoculation. Probably even a lower concentration of ChiLCV in *B. tabaci* was sufficient to make 100% transmission. However, the severity of the leaf curl symptoms in inoculated plants was less than the untreated control. The transmission of ChiLCV was decreased to 40% when *B. tabaci* were fed with 2.0 μg/mL *hsp70* dsRNA for 24 h (Figure 3e). The inoculated plants that tested positive in PCR produced mild leaf curl symptoms. Interestingly, there was no transmission of ChiLCV when *B. tabaci* was exposed to 3.0 μg/mL *hsp70* dsRNA. All the inoculated plants were without any leaf curl symptoms (Figure 3f) and tested ChiLCV-free in PCR at 35 days post-inoculation. Probably virus transmission by *B. tabaci* completely inhibited at a higher concentration of *hsp70* dsRNA. 

### 3.5. Effect of Topical Spray of hsp70 dsRNA on Mortality and Virus Transmission by B. tabaci under Semi-Field Conditions 

**Figure 4:**
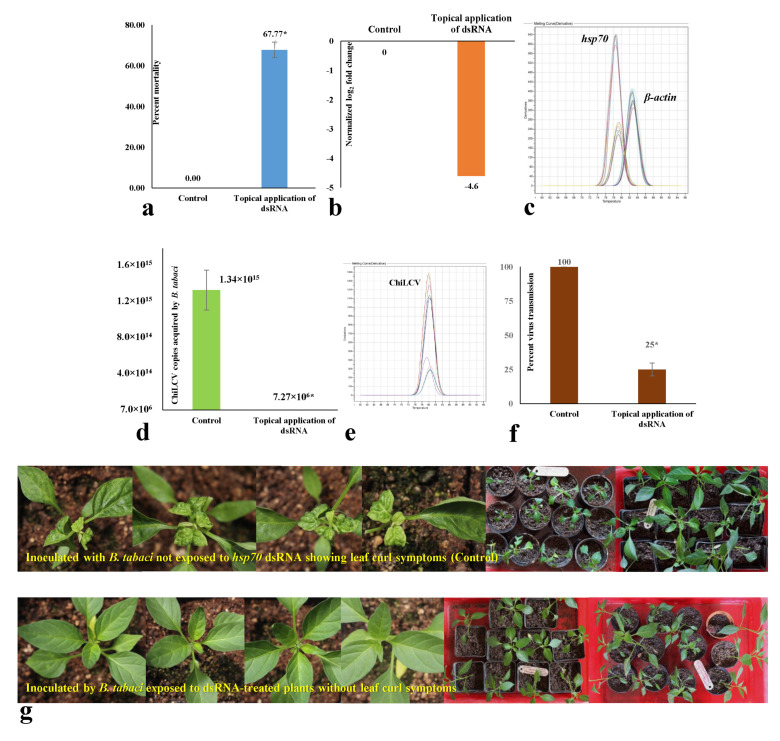
Effect of topical spray of naked *hsp70* dsRNA on mortality and ChiLCV transmission by *Bemisia tabaci*. dsRNA was not directly applied on *B. tabaci*. The naked *hsp70* dsRNA was sprayed onto the foliage of ChiLCV-infected chilli plants like an insecticide at 30 μg/mL. *B. tabaci* adults were released on the dsRNA-treated plants. (**a**) Percent mortality of adult *B. tabaci* post-24 h feeding on *hsp70* dsRNA-treated plants. The mortality in treated samples was calculated by normalizing the mortality in the control set. Values above each column are means of three replications. The error bars are standard error of the mean (SEM). Mean denoted by an asterisk (*) indicates a significant difference (*p* <  0.05). (**b**) Normalized relative expression of *B. tabaci hsp70* mRNA post-24 h feeding on *hsp70* dsRNA-treated plants in comparison to control. Relative quantification was done with respect to an endogenous control gene, *β-actin*. (**c**) Melting (dissociation) curve analysis of RT-qPCR. The specific peaks of *hsp70* and *β-actin* amplicons without any secondary peaks indicated the specificity of the reactions. (**d**) Mean ChiLCV copies retained by *B. tabaci* post-24 h exposure to ChiLCV-infected plants that were topically sprayed with *hsp70* dsRNA. (**e**) Melt curve of ChiLCV amplicon in qPCR analysis. The specific melting temperature for ChiLCV product was around 81 °C. (**f**) Transmission of ChiLCV in healthy chilli plants by *B. tabaci* post-24 h exposure to *hsp70* dsRNA-treated plants. (**g**) Leaf curl symptoms on chilli (var. Preeti) plants after 42 days of ChiLCV inoculation by *B. tabaci* exposed to *hsp70* dsRNA-treated plants in comparison to control. All the control plants produced severe leaf curl and vein-clearing symptoms. Plants that were inoculated by *B. tabaci* exposed to *hsp70* dsRNA-treated chilli plants did not produce any symptoms up to 50 days post-inoculation. A total of 5 biological replicates containing 12 plants each were used.

In a contained experiment, the efficacy of topically sprayed naked *hsp70* dsRNA was tested under semi-field atmospheric conditions. The daily temperature fluctuated between 20° and 35 °C with 40–60% RH. dsRNA was not directly delivered to *B. tabaci*. Instead, it was sprayed on ChiLCV-infected chilli plants, and *B. tabaci* adults were released on the sprayed plants. Considering the exogenous degradation of dsRNA under natural atmospheric conditions, a ten-fold higher dose of dsRNA (30 μg/mL) than the controlled condition assay was used. A 67.77% mortality of adult *B. tabaci* was recorded post-24 h exposure to dsRNA-treated plants (Figure 4a). A 4.6-fold decrease in *hsp70* mRNA expression levels of *B. tabaci* exposed to dsRNA-treated plants was also noted in RT-qPCR with respect to the endogenous control gene, *β-actin* (Figure 4b). Specific peaks of *hsp70* and *β-actin* in the melting curve analysis without any secondary peaks confirmed the specificity of the reactions (Figure 4c).

The surviving *B. tabaci* adults post-exposure to dsRNA-treated plants were further assessed for ChiLCV acquisition and transmission efficiency. A 1.84 × 10^8^-fold decrease in ChiLCV copies was observed in *B. tabaci* post-24 h exposure to ChiLCV-infected plants that were sprayed with *hsp70* dsRNA at 30 μg/mL (Figure 4d). The melt curve of ChiLCV amplicon in qPCR produced specific peaks without any secondary peaks that confirmed the specificity of the reactions (Figure 4e). When a portion of the same *B. tabaci* population was used for inoculation of ChiLCV in healthy chilli plants, a 75% decrease in transmission efficiency was confirmed in PCR test (Figure 4f) with no visible symptoms in the inoculated plants up to 50 days post-inoculation (Figure 4g). Probably, the virus titer was too low in inoculated plants to produce strong visible symptoms, whereas 100% transmission and severe leaf curl symptoms were noted in plants inoculated by unexposed *B. tabaci*. No transmission of ChiLCV was recorded in the plants mock-inoculated with untreated virus-free *B. tabaci*.

### 3.6. Stability and Persistent Efficacy of hsp70 dsRNA in Eradicating B. tabaci Population 

**Figure 5:**
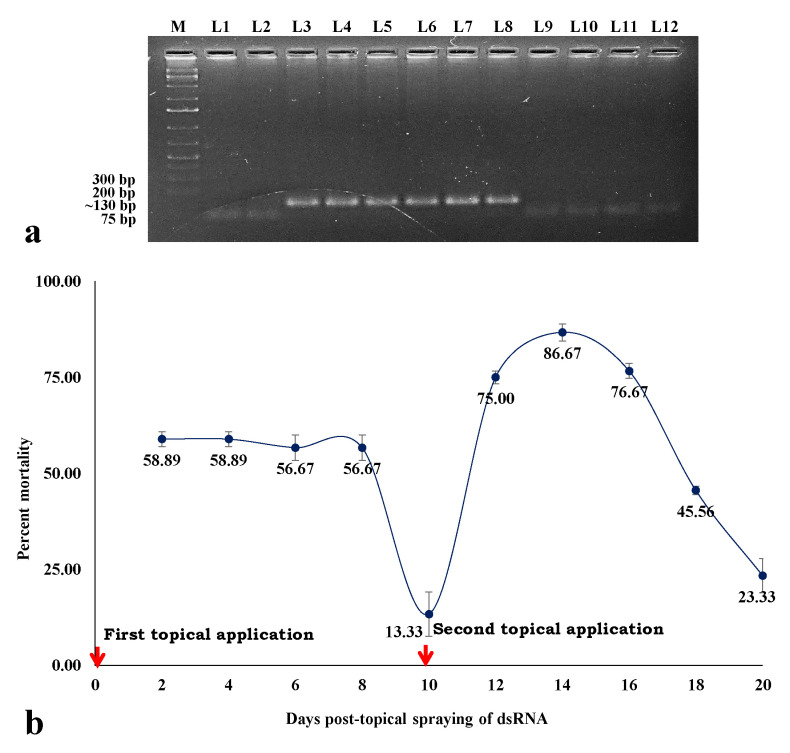
Stability and persistent efficacy of *hsp70* dsRNA. (**a**) Detection of dsRNA in RT-PCR. M: 100 bp plus DNA ladder, L1–2: untreated control, RT-PCR amplicons of dsRNA in leaf tissue 1 h (L3–4), 3 h (L5–6), 6 h (L7–8), 24 h (L9–10), and 48 h (L11–12) post-application. (**b**) Mean percent mortality of adult *B. tabaci* exposed to *hsp70* dsRNA-treated chilli plants. A total of 30 *B. tabaci* adults per plant were released at 24 h intervals, and mean percent mortality was calculated by normalizing the mortality in the control set. Values below each dot and bars on the dots indicate mean percent mortality of three replications and standard error of the mean (SEM), respectively. Each replicate contained five plants. Red arrows indicate the dates of spraying.

The stability of *hsp70* dsRNA in leaf tissue was assessed through RT-PCR using *hsp70* specific primers. A specific amplicon of ~130 bp was produced from the leaf tissues collected 1, 3, and 6 h post-spray of *hsp70* dsRNA (Figure 5a). 

To check the persistency in eradicating *B. tabaci* population by the topical spray of *hsp70* dsRNA under contained semi-field conditions, fresh *B. tabaci* adults were released on the dsRNA-treated chilli plants at 24 h intervals. After the first topical spray, mortality of 58.89% was recorded at 2 days. Significant mortality (56.67%) persisted up to 8 days after the first spray (Figure 5b). The mortality decreased to 13.33% at 10 days after the first spray. A second spray was done on the 10th day to check the effect of multiple sprays. After the second topical spray of dsRNA, the mortality percentage again rose to 75% at two days. The highest mortality (86.67%) of *B. tabaci* was recorded 4 days after the second spray. Significantly higher mortality was sustained up to 8 days after the second application and thereafter started decreasing. Overall, 2 consecutive sprays provided substantial protection against fresh releases of *B. tabaci* for up to 20 days.

## 4. Discussion

Several management strategies have been adopted around the world, with insecticides as the core component for the management of the *B. tabaci*-ChiLCV complex. However, the use of chemical insecticides has led to several detrimental effects like the development of insecticide-resistant *B. tabaci* population, destruction of beneficial organisms, environmental contamination, and health hazards [50,51,52]. There is a need for an alternative eco-friendly approach in managing *B. tabaci*-ChiLCV. RNAi or post-transcriptional gene silencing (PTGS) was introduced to study the functionality of specific genes through knocking down target gene mRNA [53]. However, in no time, it gained recognition as a potential therapeutic agent to control insect pests [54,55]. With the advancements in next-generation sequencing and transcriptomics technology, several genes have been identified as potential targets to control *B. tabaci* and its viruses [33,34,35,36,37,38,39,40,56,57,58]. However, most studies are limited to the oral feeding of the dsRNA to *B. tabaci* through artificial diet or genetic modification of the host plants [40,43,59,60]. None of these RNAi molecules have been tested for their efficacy or found effective under field conditions, limiting their large-scale adoption. RNAi using dsRNA constructs is not suitable for field conditions due to rapid degradation and a lack of reliable delivery agents. In the present study, for the first time, we demonstrated the efficacy of a naked dsRNA against an insect pest by spray-on application under semi-field conditions. The dsRNA construct targeting the *hsp70* gene of *B. tabaci* not only induced mortality in *B. tabaci* but also inhibited the transmission of ChiLCV from infected to healthy chilli plants by *B. tabaci*. 

*Hsp70* and *fas2* were found to be significantly upregulated in viruliferous *B. tabaci* along with several other genes [30,35,57]. Gene regulatory networking showed these genes were enriched with higher degrees of interactions [35]. *Hsp70* transcripts increased upon ingestion of tomato yellow leaf curl virus (TYLCV) and squash leaf curl virus (SLCV) in DNA microarray [31]. The role of *hsp70* in begomovirus transmission is well studied [30,31,57,60,61]. TYLCV CP and Hsp70 interacted in vitro and co-localized within midgut epithelial cells [30,31]. The *fas2* mRNA expression level was upregulated in viruliferous *B. tabaci* by 2.6 and 4.518-fold in the RNA-Seq and RT-qPCR assay, respectively. The Fas2 orthologue in mammals (NCAM) is known to serve as a receptor for the rabies virus [39]. Hence, we targeted to knock down these two genes, *hsp70* and *fas2,* to induce immunity to ChiLCV infection in *B. tabaci*. Conserved 128 and 150 nt stretches in the *hsp70* and *fas2* genes, respectively, were found to be specific to *B. tabaci* without any cross-reactivity to humans, mice, birds, butterflies, bees, ants, and plants. Each produced a putative siRNA of 21 nt. dsRNA was synthesized by in vivo transcription to harvest large quantities of dsRNA at a low cost. Bacterially expressed dsRNA is economical and convenient, as reported in several studies [62,63,64]. Before evaluating the efficacy of topically sprayed dsRNA, we tested it under controlled laboratory conditions. Both *hsp70* and *fas2* dsRNA were orally delivered to *B. tabaci* adults by mixing with an artificial diet in a feeding setup. Similar types of artificial feeding setup were reported to be efficient in the oral delivery of dsRNA [40,41,42]. A significant knockdown of the *B. tabaci* population was recorded at 24 h, which gradually increased with an increase in dsRNA concentration. Up to 82.22% mortality of *B. tabaci* was recorded after 24 h feeding on 3.0 μg/mL *hsp70* dsRNA. Similarly, *fas2* dsRNA at 3.0 μg/mL caused 72% mortality of *B. tabaci* 24 h post-feeding. The dsRNA exposure downregulated the *hsp70* and *fas2* mRNA expressions by 12.85- and 8.53-fold, respectively. The expression of an endogenous control gene was unregulated post-dsRNA exposure. This indicated the specificity of the dsRNA constructs to target genes. Hsp belongs to the multifunctional molecular chaperone family involved in the aggregation of damaged proteins, transportation, assembly, and disassembly of multi-structured units under stressed conditions [36,65]. The mortality of *B. tabaci* post-dsRNA feeding may be due to the loss of function via depletion of *hsp70* mRNA, which might interrupt the normal biological processes in *B. tabaci*. In *Drosophila melanogaster*, Fas2 is involved in synaptic development and growth [37,38]. It is also known to function in intracellular signaling pathways that involve mitogen-activated protein kinase (MAPK) and regulate intracellular calcium levels [38]. However, the function of *fas2* in *B. tabaci* is uncharacterized. Kanakala et al. [60] reported twisting of wings in *B. tabaci* upon silencing *hsp70*. However, we did not record any morphological deformities in treated *B. tabaci*. *Hsp70* and *fas2* might not be involved in the morphogenesis of *B. tabaci,* or longer exposure to dsRNA is required to produce such deformities. 

The persistent circulative transmission of ChiLCV by *B. tabaci* indicates that the virus particles reach the midgut after ingestion. The virions cross the midgut barrier to become circulative in the hemolymph and accumulate in the primary salivary glands [30]. *Hsp70,* along with other molecular chaperones, plays an important role in the translocation of viral proteins besides its function in viral replication, assembly, and disassembly of the viral proteins inside the hosts [66,67,68,69,70]. *Hsp70* has been found to interact with TYLCV CP in vitro and co-localize within midgut epithelial cells [31]. In the present study, oral delivery of *hsp70* dsRNA significantly reduced ChiLCV copies in *B. tabaci* Asia II 1 and its transmission to healthy chilli plants as well. The resistance to ChiLCV in *B. tabaci* was enhanced at higher concentrations of *hsp70* dsRNA. ChiLCV copies in *B. tabaci* were imperceptible when *B. tabaci* fed on 3 μg/mL *hsp70* dsRNA for 24 h. The transmission of ChiLCV from infected to healthy plants by *B. tabaci* Asia II 1 was completely inhibited at 3 μg/mL *hsp70* dsRNA treatment under controlled conditions. Our findings are consistent with Kanakala et al. [60], who reported the deleterious effect of *B. tabaci hsp70* dsRNA on TYLCV transmission. The TYLCV titer decreased in *B. tabaci* Middle East Asia Minor 1 (MEAM 1) after feeding on *hsp70* dsRNA-expressing tomato plants. Transmission of TYLCV also dropped by 12% post-silencing of *hsp70* [60]. In the present study, the inability of *B. tabaci* Asia II 1 to retain and transmit ChiLCV post-silencing of *hsp70* indicates that it might also play an important function in ChiLCV infection. However, Gotz et al. [31] reported an increase in TYLCV transmission upon blocking Hsp70 by anti-Hsp70 antibody in *B. tabaci* MEAM1. It was hypothesized that the Hsp70-TYLCV interaction mediates the degradation of virions. Downregulation of *hsp70*, *hsp40*, and *hsp20* resulted in 3.1-, 1.5-, and 1.2-fold increases in cotton leaf curl virus (CLCuV) titer within *B. tabaci*. Significantly increased transmission efficiency of CLCuV was noted in *B. tabaci* Asia II 1 when *hsp70* was silenced [61]. The difference may be due to the variation in virus species and *B. tabaci* cryptic species. The interactions of *B. tabaci* with begomoviruses are not conserved and alter with cryptic species of *B. tabaci* and begomovirus species [71,72]. *B. tabaci* MEAM1 has two genes (Bta03000 and Bta02903) annotated as *hsp70,* and their amino acid sequences differ by 97.55% [56]. They might also exhibit differential responses to begomovirus infection. Unlike *hsp70* dsRNA treatment, an increase in the ChiLCV copies in *B. tabaci* was recorded post-*fas2* dsRNA feeding. The Fas2 orthologue in mammals (NCAM) is known to serve as a receptor for the rabies virus. Although NCAM promotes the penetration of the virus in cells, it suppresses virus replication via induction of Interferon-ß [39], which is mainly involved in innate immunity against viral infection. Upregulation of *fas2* transcripts in *B. tabaci* post-ChiLCV infection might be due to the innate immune response against the virus infection [35]. We hypothesized that *fas2* has a negative regulatory role in ChiLCV infection, and thus, knocking down *fas2* increased the virus titer in *B. tabaci*. Hence, silencing *fas2* was not considered to induce resistance to ChiLCV despite its efficacy in eradicating *B. tabaci*. 

Although RNAi has emerged as a promising alternative to suppress crop pests [73,74], there are limitations in the application of dsRNA as a ‘spray-on’ technology for field use. Reduced stability in the extracellular environments of dsRNA contributes to poor RNAi response. The naked dsRNA quickly degrades in higher temperatures and UV radiation under natural environmental conditions. Besides, several insect species, mainly lepidopteran insects, have been observed to be refractory to RNAi due to degradation and poor intracellular transport of exogenous dsRNA [75,76]. Nucleases are the major factors limiting the efficacy of dsRNA [75,76,77]. In the first phase of the study, *hsp70* dsRNA showed high potential in eradicating *B. tabaci* and inhibiting ChiLCV transmission. In the next phase, the dsRNA construct was assessed under natural environmental conditions by spraying like an insecticide. Adult *B. tabaci* are highly mobile, so spraying on adult flies was not preferred. Hence, naked *hsp70* dsRNA without any delivery agent was topically sprayed onto the foliage of the ChiLCV-infected chilli plant under contained semi-field conditions. *B. tabaci* adults were released on the dsRNA-sprayed plants to evaluate the dsRNA’s efficacy in eradicating *B. tabaci* and inhibiting the transmission of ChiLCV. Considering the extracellular degradation of dsRNA and delivery through the host plant, a 10-fold higher dose than the controlled assay was used for topical application. A 67.77% mortality with a 4.6-fold decrease in *hsp70* mRNA levels in *B. tabaci* exposed to the dsRNA-sprayed plants was noted after 24 h of release. Further, 1.84 × 10^8^-fold decreased ChiLCV copies and 75% reduced transmission of ChiLCV indicated the efficiency of topically sprayed *hsp70* dsRNA in managing both the virus and its vector. Although the efficacy of topically applied naked dsRNA against plant viruses is known [78,79,80], this is the first evidence of its efficacy against a hemipteran insect like *B. tabaci*. 

Topical application of dsRNA could provide resistance for 12 to 21 days against plant viruses [78,80,81,82]. In the present study, dsRNA was detected in the leaf tissues up to 6 h post-application. Significant mortality of *B. tabaci* on the treated plants persisted up to 8 days and was further sustained up to 20 days with a second spray on the 10th day. Protection of the plants during the early growth stage is crucial in the management of begomoviruses. Begomovirus infection at a 5-leaf stage in tomatoes reduced yield by 95% [83]. Infection of ChiLCV at the seedling stage leads to a considerable reduction in plant height, internodal length, and fruiting. Hence, a 20-day shield by 2 consecutive sprays of naked *hsp70* dsRNA would protect the crucial phase of crop growth from the invasion of *B. tabaci* and ChiLCV and reduce yield losses. The foliar spray of *hsp70* dsRNA was effective under semi-field conditions where the daily temperature fluctuated between 20–35 °C with 40–60% RH. The efficacy and persistency of topically sprayed naked dsRNA at a wide temperature range endorse its potentiality for field uses as a spray-on technology. 

## 5. Conclusions

In conclusion, the spray-on application of naked dsRNA targeting *hsp70* of *B. tabaci* provides resistance to both ChiLCV and its vector, *B. tabaci*. This is a novel alternative to hazardous insecticides and may be further assessed on a large scale for its efficacy under real-field conditions.

## Data Availability

Sequences have been deposited to GenBank under accession numbers MT920041, MW399222, MZ158306, MZ766125, and OM513903.
